# Ultrahigh‐cell‐density heterotrophic cultivation of the unicellular green microalga *Scenedesmus acuminatus* and application of the cells to photoautotrophic culture enhance biomass and lipid production

**DOI:** 10.1002/bit.27190

**Published:** 2019-11-12

**Authors:** Hu Jin, Hu Zhang, Zhiwei Zhou, Kunpeng Li, Guoli Hou, Quan Xu, Wenhua Chuai, Chengwu Zhang, Danxiang Han, Qiang Hu

**Affiliations:** ^1^ Center for Microalgal Biotechnology and Biofuels, Institute of Hydrobiology Chinese Academy of Sciences Wuhan China; ^2^ College of Life Sciences University of Chinese Academy of Sciences Beijing China; ^3^ Research Center of Hydrobiology Jinan University Guangzhou China; ^4^ Microalgae Biotechnology Center, SDIC Biotech Investment Co., Ltd. State Development & Investment Corp. Beijing China; ^5^ The Innovative Academy of Seed Design Chinese Academy of Sciences Beijing China; ^6^ Key Laboratory of Algal Biology, Institute of Hydrobiology Chinese Academy of Sciences Wuhan Hubei China; ^7^ Laboratory for Marine Biology and Biotechnology Qingdao National Laboratory for Marine Science and Technology Qingdao China; ^8^ Beijing Key Laboratory of Algae Biomass SDIC Biotech Investment Corporation Beijing China

**Keywords:** heterotrophy, high‐cell‐density fermentation, lipid, microalgae, *Scenedesmus acuminatus*

## Abstract

Although production of biodiesels from microalgae is proved to be technically feasible, a commercially viable system has yet to emerge. High‐cell‐density fermentation of microalgae can be coupled with photoautotrophic cultivation to produce oils. In this study, by optimizing culturing conditions and employing a sophisticated substrate feed control strategy, ultrahigh‐cell‐density of 286 and 283.5 g/L was achieved for the unicellular alga *Scenedesmus acuminatus* grown in 7.5‐L bench‐scale and 1,000‐L pilot‐scale fermenters, respectively. The outdoor scale‐up experiments indicated that heterotrophically grown *S*. *acuminatus* cells are more productive in terms of both biomass and lipid accumulation when they are inoculated in photobioreactors for lipid production as compared to the cells originally grown under photoautotrophic conditions. Technoeconomic analysis based on the pilot‐scale data indicated that the cost of heterotrophic cultivation of microalgae for biomass production is comparable with that of the open‐pond system and much lower than that of tubular PBR, if the biomass yield was higher than 200 g/L. This study demonstrated the economic viability of heterotrophic cultivation on large‐scale microalgal inocula production, but ultrahigh‐productivity fermentation is a prerequisite. Moreover, the advantages of the combined heterotrophic and photoautotrophic cultivation of microalgae for biofuels production were also verified in the pilot‐scale.

## Graphical abstract

Jin and coworkers optimized heterotrophic culturing conditions for the unicellular alga *Scenedesmus acuminatus*, and developed the sophisticated substrate feed control strategy enabling the maximum biomass concentration of 286 and 283.5 g L^−1^ in 7.5‐L bench‐scale and 1,000‐L pilot‐scale fermenters, respectively. The authors observed that the heterotrophically grown algal cells produced more lipids than the photoautotrophic counterpart in the pilot‐scale photobioreactors. This study demonstrated the economic viability of heterotrophic cultivation on large‐scale microalgal inocula production, but ultra‐high‐productivity fermentation is prerequisite.

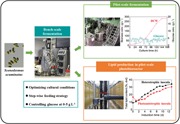

## 1 INTRODUCTION

Microalgae is a promising biomass feedstock for renewable energy production (Hu et al., 2008). Biodiesels derived from the oil‐enriched microalgal biomass have the potential to meet the increasing global demand for transport fuels by displacing fossil diesels (Chisti, 2007; Chisti, 2008). Although production of biodiesel from microalgae is proved to be technically feasible, a scalable and commercially viable system has yet to be developed (Han et al., 2012; Uduman, Qi, K. Danquah, M. Forde, & Hoadley, 2010).

Many microalgae can utilize organic carbon for cellular growth in the dark. This feature enables growing algae in fermentor to produce biomass and bioproducts. Commercial production of protein supplements and docosahexaenoic acid by using *Chlorella* and *Crypthecodinium*, respectively, has been attained in industrial‐scale fermenters (Barclay, Apt, & Dong, 2013). Recently, attempts to further extend the commercial potential of heterotrophic *Chlorella* have been focused on producing biodiesels (Miao & Wu, 2004; Xiong, Li, Xiang, & Wu, 2008). Fermentation offers many advantages in terms of biomass production, including higher cell growth rate, better control of culture conditions, and less chance of microbial contamination as compared to photoautotrophic cultivation. Thus, a cultivation strategy combining heterotrophic and photoautotrophic culture modes, which takes advantages of both the high efficiency of the former in biomass production and the low cost of the latter in oil production, has been proposed and employed for various algal species to produce biofuels (Fan et al., 2012; Han et al., 2012; Zheng, Chi, Lucker, & Chen, 2012). Although the coupled heterotrophic and photoautotrophic cultivation mode has been studied for many microalgae on different scales, its economic viability remains to be assessed, especially when utilized for biofuel production.

Fermentation, a costly and energy‐intensive process, will become economically feasible only if ultrahigh‐cell density can be achieved. To date, the reported maximum biomass concentration/productivity of most oleaginous microalgae under heterotrophic culture are still not as competitive as that of the other industrial microorganisms (e.g., bacteria or yeast). Besides, the inherent longer doubling time of microalgae, and the lack of effective growth condition optimization as well, have limited their full growth potential. It remains elusive regarding whether heterotrophically grown algal cells could adapt to the stress environmental conditions used for inducing lipid production, such as high‐light and nitrogen depletion conditions, after shifting from heterotrophic to photoautotrophic cultivation mode.

A *Scenedesmus acuminatus* strain that shows potential in heterotrophic growth and lipid production was obtained in a previous study (Wang, Sun, Li, & Zhang, 2014). Though this *S*. *acuminatus* strain accumulated little lipid under heterotrophic conditions, the heterotrophically grown cells can be used as inoculum for lipid production under photoautotrophic conditions. Thus, this study aimed to improve the biomass production by optimizing heterotrophic culture conditions and process control means, and to compare their capabilities in lipid production with photoautotrophically grown cells in pilot‐scale. To evaluate the economic feasibility of heterotrophic cultivation, the technoeconomic (TE) analysis for both heterotrophic and photoautotrophic processes were conducted by utilizing the pilot‐scale experimental data. Based upon the results of TE analysis, this study demonstrated the economic viability of the ultrahigh‐cell‐density fermentation in the entire chain of algal biofuel production.

## 2 MATERIALS AND METHODS

### 2.1 Algal strain and growth conditions

*S. acuminatus* GT‐2 was isolated from South Lake of Guangzhou, China. Algal cells were maintained in a modified Endo growth medium, containing 30 g/L glucose, 3 g/L KNO_3_, 1.2 g/L KH_2_PO_4_, 1.2 g/L MgSO_4_•7H_2_O, 0.2 g/L trisodium citrate, 0.016 g/L FeSO_4_•7H_2_O, 2.1 mg/L EDTA‐Na_2_, 0.03 g/L CaCl_2_•2H_2_O, 2.86 mg/L H_3_BO_3_, 0.222 mg/L ZnSO_4_•7H_2_O, 1.81 mg/L MnCl_2_•4H_2_O, 0.021 mg/L Na_2_MoO_4_, and 0.07 mg/L CuSO_4_•2H_2_O. To prepare the inoculants for fermentation, a single colony of *S*. *acuminatus* GT‐2 was inoculated into 100 ml of modified Endo medium in a 250‐ml Erlenmeyer flask and grown at 30°C for 5–6 days in a shaking incubator at the speed of 180 rpm, which was then used as inoculum for fermentation.

Bench‐scale fermentation experiments were performed in a 7.5‐L bioreactor (BioFlo & CelliGen 310; New Brunswick) with the initial working volume of 2.8 L. The pH was maintained automatically by the addition of 3 M NaOH or 1 M HCl solution. Aeration was maintained at 1 vvm with the airflow rate of 2.8 L/min. Dissolved oxygen (DO) was controlled automatically above 40% by coupling with the stirring speed. In fermentor batch medium, KNO_3_ was replaced by 0.84 g/L urea. Feeding medium used during fermentation process was the 25‐fold concentrated batch medium, containing 750 g/L of glucose.

Pilot‐scale fermentation was carried out in a 1,000 L stirred tank bioreactor (WKT 1000 L; Yangzhong Weikete Biological Engineering Equipment Co., Ltd., China) containing 300 L medium. To shorten the culture period, the 1,000‐L pilot‐scale fermentation was inoculated with 40 L high‐cell‐density culture (80 g/L) from the 100‐L bioreactor after 4 days’ fed‐batch cultivation, which was inoculated from 7.5‐L bench‐scale fermentor. The initial biomass concentration in 1000‐L fermentor cultivation was approximately 10 g/L. For 1000‐L pilot‐scale fermentation, the aeration rate and the agitation speed was initially set at 20 m^3^ h^−1^ (1 vvm) and 80 rpm, respectively. The pressure of the inner bioreactor was kept at 0.035 Mpa.

For photoautotrophic culture, the growth medium BG‐11 was used (Rippka, Deruelles, Waterbury, Herdman, & Stanier, 1979). Algal cells were grown in 750 ml BG‐11 in an 800‐ml column PBR (i.d. 5 cm) under continuous light (250 μmol·m^−2^·s^−1^) at 25 ± 2°C. Mixing and aeration were provided by bubbling air containing 2.0% (v/v) CO_2_. The cell culture was sequentially scaled‐up to a 12‐L panel PBR and a 380‐L tubular PBR followed by a 1,300‐L tubular PBR. The inoculum size during each step was 10% (v/v) of the total volume of culture media.

### 2.2 Induction of lipid production

Pilot‐scale lipid production experiments were conducted in a 5,300‐L tubular PBR (i.d. 5 cm) outdoors from June to September in 2016 (39° 97′ N 117° 06′ E, Sanhe, China). Algal cells grown in 7.5‐L fermentor and 1,300‐L PBR indoors were transferred to two parallel 5,300‐L PBR and induced for lipid production in the N‐limited BG‐11 medium containing 1.1 mM nitrate for 13 days. For the outdoor experiments, CO_2_ was injected into the culture during daylight hours to maintain pH in the range of 6.5 to 6.8. The cooling system prevented the culture temperature from exceeding 35°C. During the night, the culture temperature was allowed to equilibrate to ambience.

### 2.3 Analytical procedures

Cell growth was monitored by measuring the dry biomass weight according to Chini Zittelli, Pastorelli, and Tredici (2000). The cell number was counted using a haemocytometer after appropriate dilution. The glucose concentration was determined with a Safe‐Accu UG Blood Glucose Monitoring System (Model BGMS‐1; Sinocare Inc., Changsha, China). The contents of total lipids were determined according to the method described in a previous study (Jia et al., 2015).

### 2.4 Technoeconomic analysis

The cost of heterotrophic cultivation for inoculum production was compared to that of conventional photoautotrophic culturing modes, including open‐pond and PBR systems, both of which are widely used in algal industries and were thus used as references to evaluate the economic feasibility of heterotrophic cultivation here. The key input assumptions for TE analysis are summarized in Tables S1 and S2. To evaluate the effect of scale on production cost, it was assumed that two different inoculum production capacities are 1,000 and 10,000 tons per year on 300 operating days, respectively. A set of tubular photobioreactor for the imocula production is 98 m^3^, and the culture volume of the open pond was assumed to be 1,000 m^3^. According to our pilot‐scale experimental results in the 1,000‐L fermentor, we assumed the average harvest biomass concentration in a 120 m^3^ inocula production fermentor is 200 g/L, achieved within 10 days in a batch of the production process. The initial biomass concentrations in open pond and tubular photobioreactor are 0.1 and 0.2 g/L, and their harvest biomass concentrations are 0.8 and 2 g/L, respectively. The collapse rate caused by biotic contamination in heterotrophic fermentation, open‐pond, and tubular photobioreactor cultivations was assumed to be 10%, 15%, and 5%, respectively. For economic assumptions, all the capital and operating costs for both heterotrophic and photoautotrophic inocula production were estimated based on vendor quotes, previous studies, or standard engineering estimates. The biomass cost was calculated based on the model reported by Tapie and Bernard (1988). The cost structure includes the following two major parameters: capital investment costs and operating costs. The operating costs include fixed costs (e.g., labor, overhead, and maintenance) and variable costs (e.g., nutrients, power, CO_2_, and water). For heterotrophic and photoautotrophic culture, overhead is 60% and 2% of the installed equipment cost for labor and maintenance, respectively. The lifetime of open‐pond and tubular PBR was assumed to be 10 and 15 years, respectively. For photoautotrophic culture, the CO_2_ was assumed to be supplied from a nearby power plant. Water was recycled in photoautotrophic culturing systems, and the evaporation rate of water in open pond was assumed to be 1 cm/day.

### 2.5 Statistical analysis

The values are expressed as mean ± standard deviation. The statistical tests were performed by using one‐way analysis of variance in SPSS (version 19.0). Statistically significant difference was considered at *p* < .05.

## 3 RESULTS

### 3.1 Optimization of heterotrophic culturing conditions for *S. enedesmus acuminatus* GT‐2

**Figure 1:**
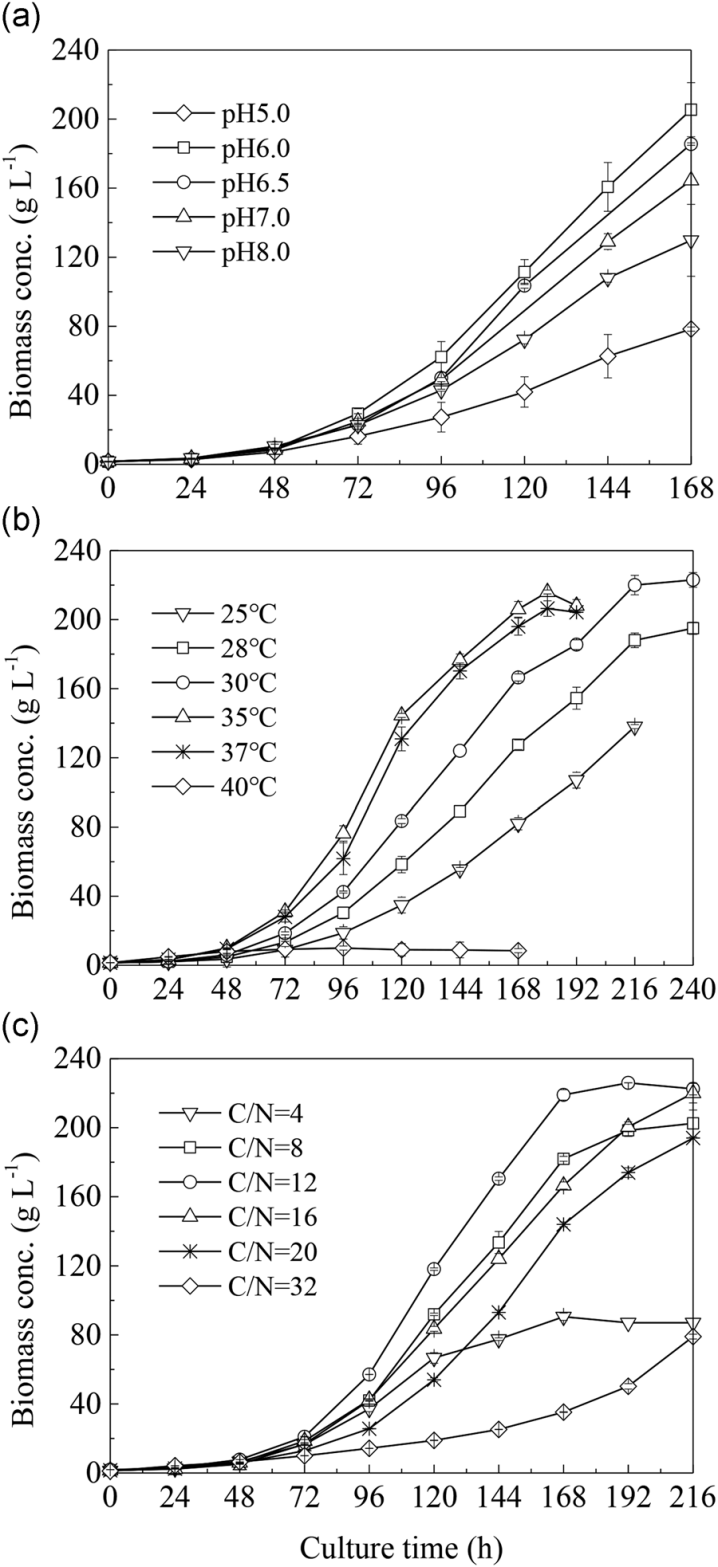
Effects of different pH (a), temperatures (b), and C/N ratios (c) on *Scenedesmus acuminatus* cellular growth under heterotrophic conditions in 7.5‐L bioreactors

The effect of pH on heterotrophic growth of *S*. *acuminatus* GT‐2 was investigated in 7.5‐L fermentors, in which the culture pH was maintained at 5.0–8.0 by using the pH‐stat mode. As shown in Figure 1a, *S. acuminatus* GT‐2 favored a weak acidic and neutral pH environment. Under the optimum pH of 6, the highest biomass concentration (205.4 g/L) was achieved at the end of fermentation (168 hr). Thus, the following optimization experiments were conducted at pH 6.0.

When the culture temperature was increased from 25°C to 30°C, *S*. *acuminatus* GT‐2 grew more rapidly, reaching the highest cell density of 223 g/L at 30°C after 240 hr (Figure 1b). Further increase in growth occurred when raising the temperature to 35°C and 37°C during the first 168 hr, after which the cellular growth leveled off and then declined. When the temperature was increased to 40°C, little growth was observed (Figure 1b). Although it took less time to achieve the highest biomass yield at 35°C or 37°C as compared to at 30°C, longer period of time of imposing the maximum aeration rate (600 rpm) was required to maintain the DO level at 40% (Figure S1), which indicated more energy input. Thus, 30°C was chosen as the optimal culture temperature for the following experiments.

The C/N ratio of a culture medium is thought to be one of the most critical nutritional factors affecting microbial growth (Huang, Chen, Wei, Zhang, & Chen, 2010). As shown in Figure 1c, the C/N ratio of 12 sustained the highest growth rate and maximum final dry biomass concentration of 220 g/L.

### 3.2 A stepwise constant feeding strategy enhanced biomass production

**Figure 2:**
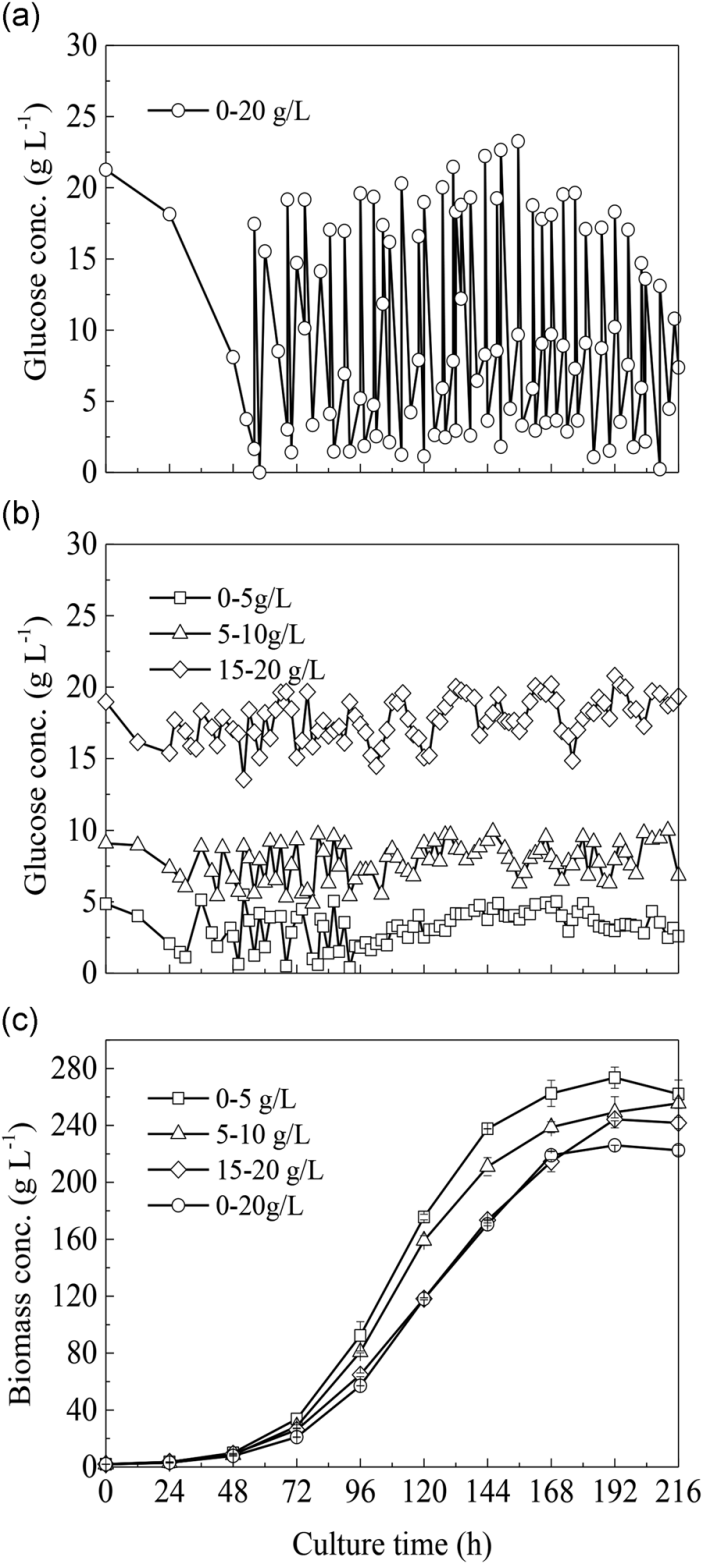
Comparison of biomass production among different glucose feeding strategies. (a) Pulsed feeding by controlling glucose concentration in the range of 0–20 g/L. (b) Stepwise constant feeding by controlling glucose concentration under different ranges of 0–5, 5–10, and 15–20 g/L. (c) Biomass concentration under different feeding strategies

Growth of *S*. *acuminatus* GT‐2 cells were compared under two different glucose supply modes, that is, the traditional pulse feeding mode and a stepwise constant feeding mode proposed in this study. For the pulse feeding mode, an upper level of glucose concentration was preset (e.g., 20 g/L). When its concentration decreased from the preset level to nearly depletion, glucose was supplied into the culture according to the preset concentration within 10 min (Figure 2a). In the proposed stepwise constant feeding mode, glucose concentrations were finely controlled at a relatively stable level (e.g., 0–5, 5–10, or 15–20 g/L; Figure 2b). Compared to the pulse feeding mode, more frequently sampling, measuring, and adjusting feeding rate are required when the stepwise constant feeding was implemented.

The results showed that the biomass concentrations were very close among all the cell cultures with different feeding strategies before 72 hr (Figure 2c). After 72 hr, the biomass concentrations obtained with the glucose controlled at relatively low concentrations and within less fluctuating range (0–5 or 5–10 g/L) were obviously higher than those controlled by using the pulse feeding (0–20 g/L), or by stepwise feeding higher glucose concentration (15–20 g/L). The highest biomass concentration reached 273.5 g/L at 192 hr when the glucose concentrations were controlled at 0–5 g/L, which was 1.2‐fold higher than that obtained with the pulsed feeding strategy (226 g/L; Figure 2c). Under such an optimal growth condition, the doubling‐time and specific growth rate of *S. acuminatus* GT‐2 during exponential growth phase was 16 hr and 0.043 hr^−1^, respectively.

The biomass concentration, total glucose consumption amount, biomass productivity, and yield on glucose attained under different feeding strategies and glucose concentrations are listed in Table 1. It was shown that the feeding strategies and glucose concentration not only affected the growth and production performance of *S*. *acuminatus* cells, but also influenced the glucose‐to‐biomass conversion efficiency. Glucose‐to‐biomass conversion efficiency achieved 60.09% by stepwise constantly feeding the glucose at a steady low level of 0–5 g/L, which was significantly higher than that of the pulsed feeding mode (i.e., 55.6%; *p* < .05). The biomass yields obtained by using such a strategy were all higher than that of the pulsed feeding mode (*p* < .05). Taken together, the data indicated that the utilization of carbon source by *S*. *acuminatus* cells was highly efficient when glucose concentrations were controlled at a relatively steady and low level.

**Table 1:** Comparison of major fermentation performances among the cultures employed with different feeding strategies and glucose concentration levels

Feeding strategies	Glucose concentration controlled (g/L)	Maximum biomass conc. (g/L) & time	Final biomass conc. (g/L)	Total glucose consumed (g)	Glucose‐to‐biomass conversion (%)	Ave. productivity (g·L^−1^·h^−1^)^a^
Stepwise constant feeding	0–5	273.5 ± 7.4 (192 hr)	262 ± 9.2	2,660	60.09 ± 0.56	1.21 ± 0.01
Stepwise constant feeding	5–10	258 ± 3.5 (216 hr)	258 ± 3.5	2,242	59.20 ± 1.06	1.19 ± 0.01
Stepwise constant feeding	15–20	244 ± 3.9 (216 hr)	244 ± 3.9	1,971	58.20 ± 1.28	1.13 ± 0.02
Pulsed feeding	0–20	226 ± 3.0 (192 hr)	222.5 ± 3.5	2,024	55.60 ± 0.88	1.03 ± 0.01

^a^ Biomass productivity (g·L^−1^·h^−1^) = dX/dt = (*X*_2_−*X*_1_)/(*t*_2_−*t*_1_), where *X*_1_ and *X*_2_ are the biomass concentration at time *t*_1_ and *t*_2_ of the fermentation.

Compared to those published data, we achieved an ultrahigh‐cell density of 286 g/L, which was 2.4‐fold higher than the highest level reported up to date (Table 2). Moreover, the maximum biomass productivity of *S*. *acuminatus* reached 3.81 g·L^−1^·h^−1^, showing a great potential in commercial applications.

**Table 2:** Overview of maximum biomass concentration and productivity of microalgae under heterotrophic fed‐batch cultivation

Microalgal species	Product	Maximum biomass concentration (g/L)	Maximum biomass productivity (g·L^−1^·h^−1^)	References
*Aurantiochytrium* sp.	DHA	31.8	0.44	Ryu, Kim, Kim, Han, and Yang (2013)
*Chlorella protothecoides*	Lutein	19.6	0.11	Shi, Jiang, and Chen (2002)
*Chlorella protothecoides*	Lipids	70.9	0.39	Yan, Lu, Chen, and Wu (2011)
*Chlorella protothecoides*	Biomass	116	0.98	Wu and Shi (2007)
*Chlorella regularis*	Biomass	84	2.8	Sansawa and Endo (2004)
*Chlorella vulgaris*	Biomass	117.2	3.66	Doucha and Lívanský (2011)
*Chlorella zofingiensis*	Astaxanthin	53	0.14	Sun, Wang, Li, Huang, and Chen (2008)
*Chlorella sorokiniana*	Lipids	103.8	0.45	Zheng et al. (2013)
*Chlorococcum* sp.	Ketocarotenoid	18	‐	Zhang and Lee (2001)
*Cryptecodinium cohnii*	DHA	109	0.28	De Swaaf, Sijtsma, and Pronk (2003)
*Euglena gracilis*	α‐Tocopherol	48	0.26	Ogbonna, Tomiyamal, and Tanaka (1998)
*Galdieria sulphuraria*	Phycocyanin	109	0.72	Graverholt and Eriksen (2007)
*Galdieria sulphuraria*	Phycocyanin	116	0.34	Schmidt et al. (2005)
*Haematococcus pluvialis*	Astaxanthin	26	0.06	Wan et al. (2015)
*Neochloris oleoabundans*	Lipids	20.9	0.08	Morales‐Sánchez, Tinoco‐Valencia, Kyndt, and Martinez (2013)
*Nitzschia laevis*	EPA	40	0.12	Wen and Chen (2002)
*Scenedesmus acuminatus* GT‐2	Lipids	286	3.81	This study

Abbreviation: DHA, docosahexaenoic acid.

### 3.3 Heterotrophic cultivation of *S*. *acuminatus* GT‐2 in 1,000‐L fermentor

**Figure 3:**
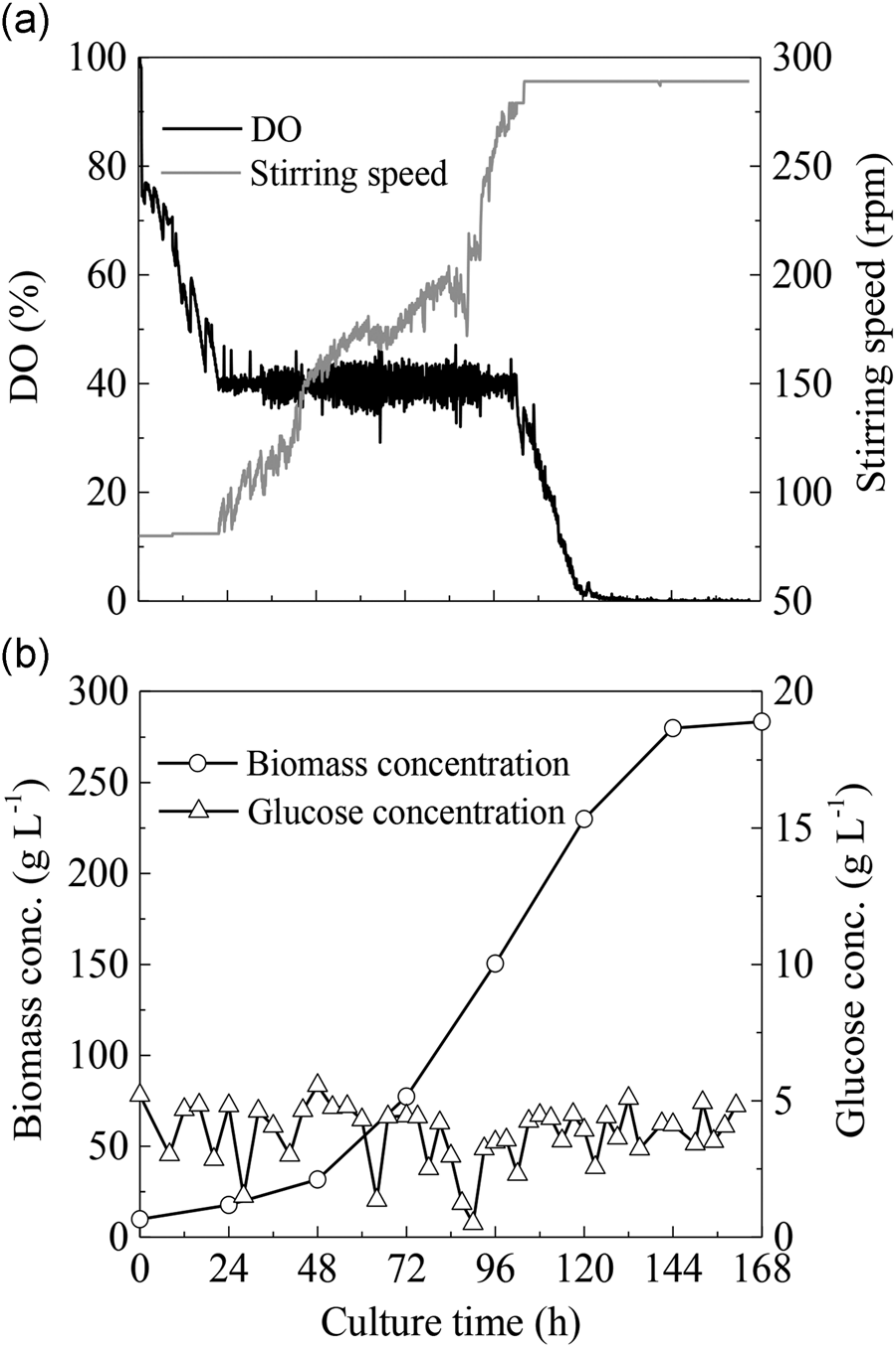
Scale‐up of heterotrophic culture of *S*. acuminatus in 1,000‐L fermentor with the optimal cultural conditions. (a) Time courses of dissolved oxygen and stirring speed. (b) Time courses of biomass and glucose concentrations

In a pilot‐scale fermentation experiment carried out in a 1,000‐L fermentor, the glucose concentration was maintained within the optimum range of 0‐5 g/L by using the stepwise constant feeding strategy. As a result, the maximum biomass concentration reached at 283.5 g/L (Figure 3b), which was very close to the highest level (286 g/L) achieved in the 7.5‐L bench‐scale fermentor. Figure 3a showed the changes of DO and stirring speed during the scale‐up cultivation. When the biomass concentration reached a high level of about 160 g/L, the DO level cannot be maintained at a constant level and gradually dropped to 0 at about 120 hr. However, it seemed that the growth of *S*. acuminatus cells was not affected by the decline of DO, and a continuous increase of biomass concentration was observed when the DO remained at 0 from 120 through 144 hr (Figure 3). In addition, the increase in inoculation density shortened the lag phase and finally reduced the whole culture period. When the initial biomass concentration increased from 2 g/L in bench‐scale fermentor to 10 g/L in pilot‐scale fermentor, the lag phase was shortened by about 24 hr, and the cell culture entered into the exponential growth stage from 24 hr after inoculation (Figures 2c and 3b).

### 3.4 Scale‐up of lipid production in outdoor 5,300 L tubular photobioreactors

To further verify the feasibility of using heterotrophic microalgal cells for lipid production and to compare its performance with the photoautotrophic cells, pilot‐scale experiments were conducted in two parallel outdoor tubular photobioreactors. One was inoculated with the algal cells from the heterotrophic late‐exponential phase in 7.5 L fermentors, and the other one used the algal cells from the photoautotrophic exponential growth phase in 1,300 L tubular photobioreactors. Three critical environmental parameters including temperature, light intensity, and pH were monitored during the cultivation period. The results showed that these parameters were very close between the two‐parallel tubular photobioreactors (Figure S2).

**Figure 4:**
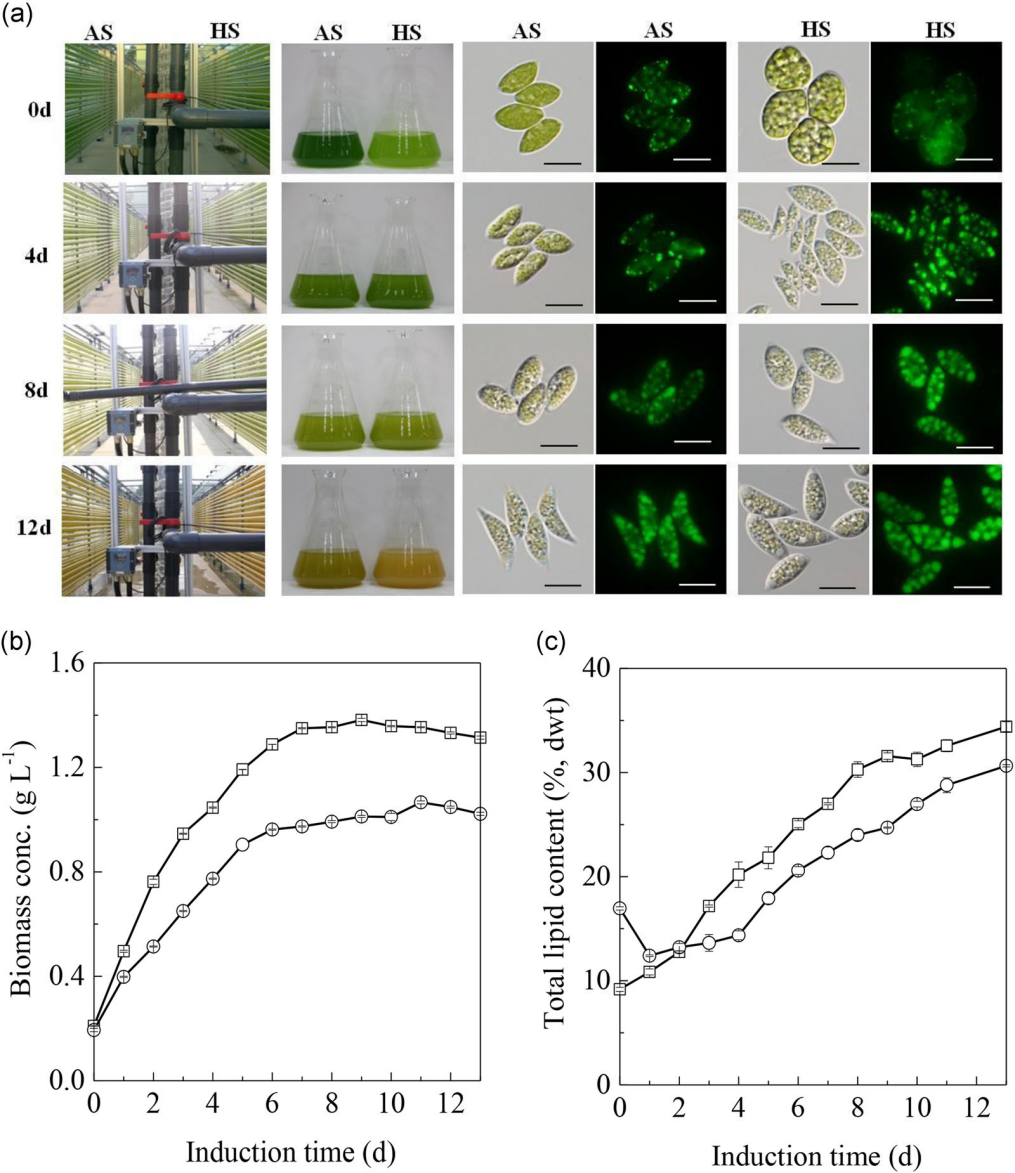
Lipid production in pilot‐scale tubular photobioreactors. (a) Culture appearance and cell morphology; (b) cell growth; (c) lipid contents of the culture inoculated with the cells from heterotrophic (□) and photoautotrophic (○) cultures. AS: Inocula from photoautotrophic culture; HS: inocula from heterotrophic culture; scale bar = 10 μm [Color figure can be viewed at wileyonlinelibrary.com]

The outdoor scale‐up experiments underlined the advantage of heterotrophically grown algal cells over the photoautotrophic counterparts in terms of lipid production, which is in accordance with the findings of previously published studies (Han et al., 2012; Zheng et al., 2012). It was observed that photoautotrophically grown algal cells contained greater amounts of chlorophylls than the heterotrophically grown algal cells, giving rise to the darker green color of the culture (Figure 4a). Under the N‐limitation conditions, the color of the two cultures turned yellow, accompanied with accumulation of neutral lipids, as indicated by BODIPY staining and microscopic observation (Figure 4a). For the large‐scale culture inoculated with the heterotrophically grown cells, the maximum biomass concentration and lipid content was 1.38 g/L and 34.4%, respectively, which was 1.3‐ and 1.1‐fold greater than that of the culture inoculated with photoautotrophically grown cells (Figure 4b,c). These results suggested that the culture mode coupling heterotrophy and photoautotrophy was more productive than the traditional photoautotrophic mode in regard to both biomass and lipid production. Under such a combined culture mode, the average lipid productivity in 5,300 L pilot‐scale photobioreactor reached 45.05 mg·L^−1^·d^−1^, which was the highest level among the reported lipid productivities of microalgae cultured on a similar scale (Table 3).

**Table 3:** Summary of lipid production performance by outdoor cultivation of microalgae on various cultural scales

Microalgal species	Type of cultural system	Inocula source & culture mode	Culture scale (L)	Lipid content (%, dwt)	Lipid Productivity (mg·L^−1^·d^−1^)	References
*Chlorella zofingiensi*	Flat plate	P and B	60	33.80	22.30	Feng, Deng, Hu, and Fan (2011)
*Chlorella* sp.	Bag PBR	P and B	120	23.00	13.70	Moheimani (2013)
*Graesiella* sp.	Raceway pond	P and B	40,000	31.80	14.50	Wen et al. (2016)
*Monoraphidium dybowskii*	Raceway pond	P and S	40,000	30.00	27.20	Yang, He, and Hu (2018)
*Nannochloropsis* sp.	Green wall panel	P and B	110	60.00	204.00	Rodolfi et al. (2010)
*Nannochloropsis gaditana*	Tubular PBR	P and C	340	18.60	110.00	San Pedro, Gonzalez‐Lopez, Acien, and Molina‐Grima (2014)
*Nannochloropsis* sp.	Green wall panel	P and B	590	43.00	110.00	Biondi et al. (2013)
*Nannochloropsis gaditana*	Raceway pond	P and C	792	25.60	30.40	San Pedro, González‐López, Acién, and Molina‐Grima (2015)
*Nannochloropsis* sp.	Raceway pond	P and B	8,000	28.00	4.69	Zhu et al. (2014)
*Scenedesmus obtusus*	Tubular PBR	P and S	500	13.40	19.00	Hulatt and Thomas (2011)
*Scenedesmus acutus*	Raceway pond	P and B	2,278	21.50	9.20	Eustance, Wray, Badvipour, and Sommerfeld (2016)
*Scenedesmus acuminatus*	Tubular PBR	P and B	5,300	31.04	27.45	This study
*Scenedesmus acuminatus*	Tubular PBR	H and B	5,300	35.68	45.05	This study

Abbreviations: B, batch culture; C, continuous culture; H, seed from heterotrophic cultivation; P, seed from photoautotrophic cultivation; S, semi‐continuous culture; T, two‐step culture.

### 3.5 TE analysis

**Figure 5:**
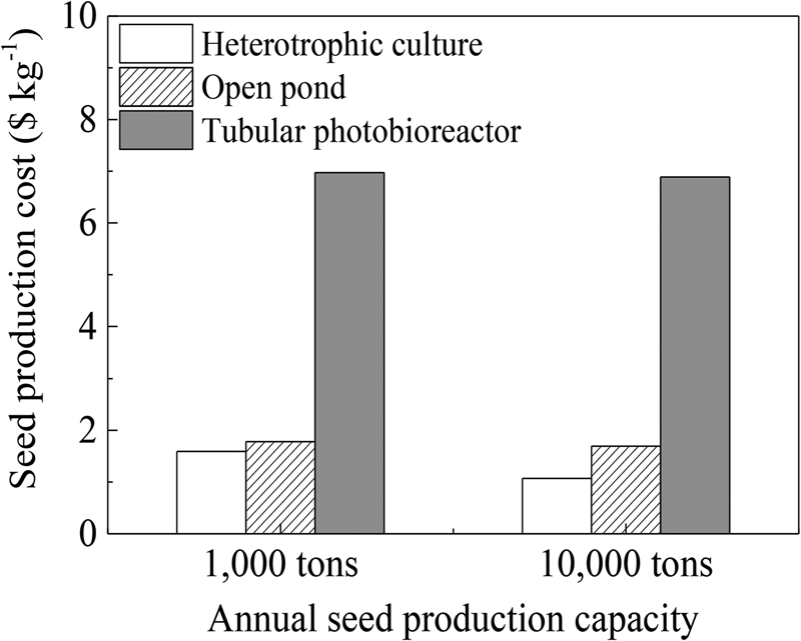
Cost comparison among different culture modes

To evaluate the economic feasibility of heterotrophic cultivation on large‐scale for inoculum production, we conducted TE analysis based on our pilot‐scale data along with several key assumptions. The cost of heterotrophic culture in fermentors was compared with that of the traditional photoautotrophic culture in open ponds and tubular photobioreactors. As shown in Figure 5, to produce the same amount of biomass for lipid production, the production cost of heterotrophic culture was the lowest among the three cultivation modes. It should be pointed out that the cost of biomass production by using the industrial‐scale heterotrophic cultivation was estimated based on a high biomass concentration of 200 g/L (Table S1), which is achievable at least for *S. acuminatus* GT‐2 and *C.sorokinian* GT‐1 (unpublished data). With the increase of annual production capacity from 1,000 to 10,000 tons, the cost can be reduced from $1.59 to $1.07 per kilo of algal biomass (dry weight equivalent) because the increased fermentor volume (from 120 to 200 m^3^) could considerably reduce both the capital and operational costs (Table 4). Due to the high capital investment (~70% of the total cost), the cost of biomass production in tubular photobioreactor was the highest among three culture modes, about five‐fold higher compared to cultivations in fermentor and open ponds (Figure 5).

**Table 4:** Comparison of the annual production cost compositions among different culture modes

No.	Items	Open pond		Tubular photobioreactor		Heterotrophic culture	
		Cost/$1 M	Percentage (%)	Cost/$1 M	Percentage (%)	Cost/$1 M	Percentage (%)
**1,000 t per year**							
1	Equipment depreciation	0.5200	29.50	4.7456	68.68	0.2658	16.75
2	Land use	0.0138	0.79	0.0120	0.17	0.0004	0.03
3	Water	0.3024	17.15	0.0224	0.32	0.0070	0.44
4	Nutrient	0.0573	3.25	0.0503	0.73	0.6224	39.23
5	Power	0.6523	37.00	1.6253	23.52	0.5989	37.75
6	CO_2_	0.0000	0.00	0.0000	0.00	0.0000	0.00
7	Maintenance	0.0000	0.00	0.2373	3.43	0.0053	0.34
8	Labor	0.2170	12.31	0.2173	3.14	0.0868	5.47
9	Total cost	1.7628	100.00	6.9099	100.00	1.5866	100.00
**10,000 t per year**							
1	Equipment depreciation	5.2354	30.91	47.6734	69.29	0.94	8.85
2	Land use	0.1374	0.81	0.1196	0.17	0.0013	0.01
3	Water	3.0018	17.72	0.2254	0.33	0.0000	0.00
4	Nutrient	0.5688	3.36	0.5072	0.74	6.2226	58.41
5	Power	6.4753	38.23	16.3760	23.80	3.3372	31.33
6	CO_2_ cost	0.00	0.00	0.00	0.00	0.00	0.00
7	Maintenance	0.00	0.00	2.3837	3.46	0.0189	0.18
8	Labor	1.5191	8.97	1.5191	2.21	0.1302	1.22
9	Total cost	16.9378	100.00	68.8045	100.00	10.6530	100.00

**Figure 6:**
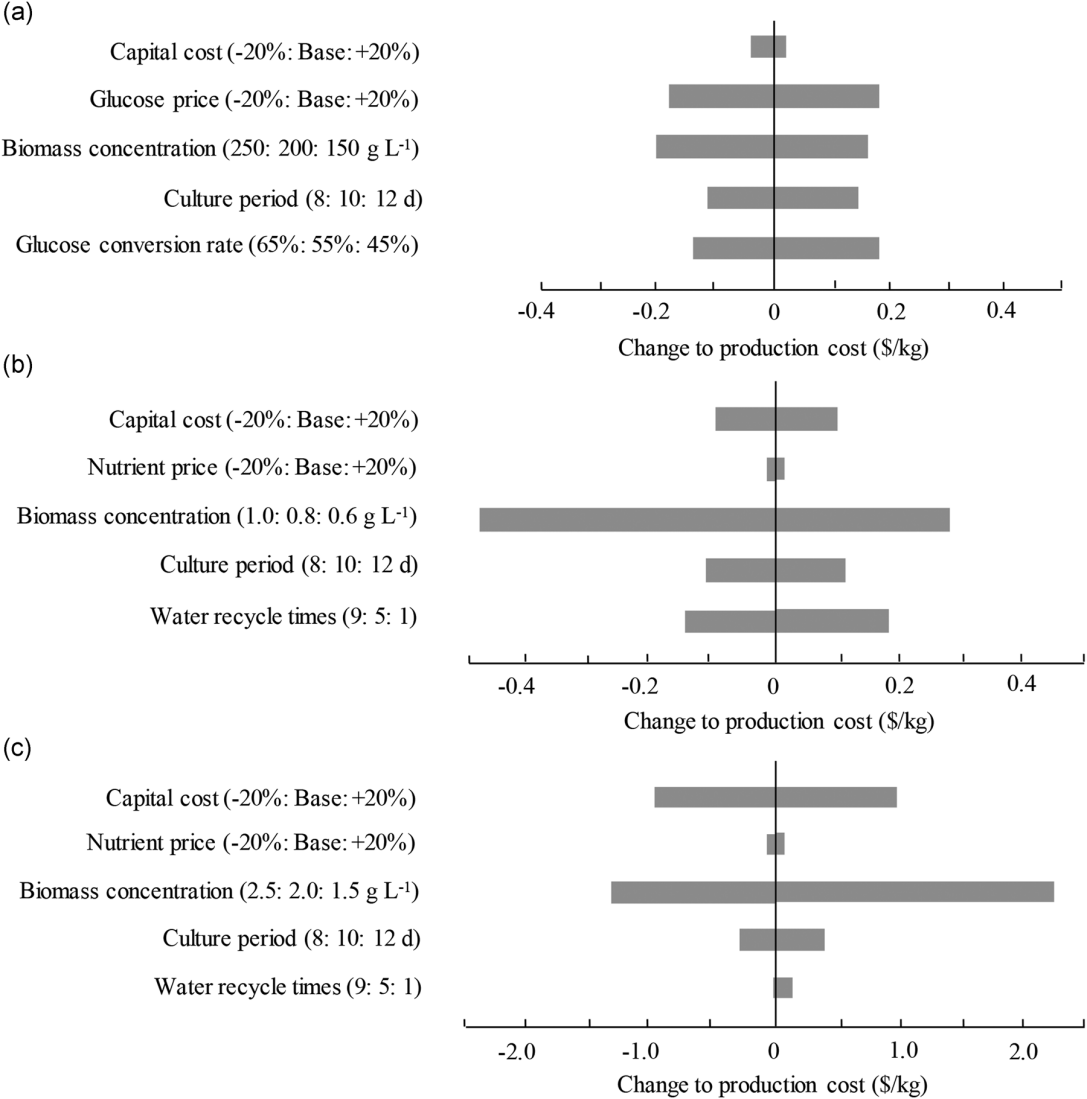
Sensitivity analysis of heterotrophic (a), open pond (b), and tubular photobioreactor (c) cultivation system

To evaluate the effect of future improvements in algal production technology and process on the overall cost, the sensitivity analysis was performed. For heterotrophic culture, the achievable maximum biomass concentration was found to have the greatest impact on the overall production cost. Moreover, the price of glucose and its conversion efficiency into algal biomass also have great influence on the cost (Figure 6a–c). Thus, the cost of heterotrophic culture could be reduced by improving the glucose conversion efficiency, and by utilizing any alternative low‐cost organic carbon sources as well. In addition to improving biomass production, the cost of open‐pond cultivation could be further reduced by increasing the times of water recycling in algal cultivation. For tubular photobioreactors, the development of low‐cost and highly efficient photobioreactors can simultaneously improve biomass productivity while reducing the capital cost.

## 4 DISCUSSION

This study reported ultrahigh‐cell‐density heterotrophic cultivation of the unicellular green alga *S*. *acuminatus* with a maximum biomass concentration of 286 and 283.5 g/L in 7.5 L bench‐scale and 1,000 L pilot‐scale fermenters, respectively. These are the highest levels in the microbial heterotrophic cultivation up to date. It is noteworthy that when the cell density of *S*. *acuminatus* was 250 g/L dry weight and above, the water content of the cells was lower than 60% (Figure S3), which is dramatically different from most cells that are known to contain approximately 80% water. Under such circumstances, the wet algal pellets occupied 55% of the culture volume, whereas the water outside the cells filled the remaining volume (ca., 45%). In addition, the viscosity of the *S*. *acuminatus* culture is not as high as that of many well‐known industrial micro‐organisms. For instance, when the dry cell density of *Pichia pastoris* is above 120 g/L, the culture broth appears slurry and hard to be stirred. In contrast, the ultrahigh‐cell‐density culture (i.e., 280 g/L) of *S*. *acuminatus* can be mixed well, which might be attributable to little extracellular polysaccharides secreted by this organism during cultivation.

This study demonstrated the coupled heterotrophic and photoautotrophic culturing mode is a promising technology for microalgal biodiesel production when ultrahigh‐cell‐density heterotrophic cultivation is achieved through employing a sophisticated substrate feed control strategy. Such a technology was developed through two‐tier optimization. First, the traditional pulse addition of glucose was replaced by a stepwise constant feeding strategy, which obviously increased the biomass productivity of *S*. *acuminatus* in fed‐batch culture. Pulse addition of carbon sources has been employed in heterotrophic cultivation for many microalgae, especially for *Chlorella* strains. However, either dramatic fluctuation or depletion of substrate could cause stress to microalgal cells. For example, it has been reported that the microalga *G*. *sulphuraria* metabolized intracellular components when the sugar is starved (Schmidt, Wiebe, & Eriksen, 2005). By utilization of the stepwise constant feeding strategy, the concentration of glucose was maintained at a relatively steady level to avoid adverse effect caused by the periodic limitation and starvation of glucose.

Second, the highest average growth rate (1.21 g·L^−1^·h^−1^) and glucose conversion efficiency (60.09%) were achieved by maintaining the glucose at a relatively low constant concentration (<5 g/L). It has been observed in a number of microalgal heterotrophic culture that keeping low concentration of glucose is essential for sustaining a higher specific growth rate. For example, a glucose concentration of 2.5 g/L is optimum for the growth of *C*. *saccharophila*, whereas inhibition occurred at the concentration >25 g/L (Tan & Johns, 1991). For an eicosapentaenoic acid‐producing diatom *Nitschia laevis*, the growth rate decreased with the increase of glucose concentration from 1 to 40 g/L (Wen & Chen, 2000). In the green alga *C*. *kessleri*, glucose uptake is mediated by a hexose/H^+^ symporter, which shows saturation of behavior with a Km value of about 0.3 mM (Komor, Schwab, & Tanner, 1979). This finding can explain why very low concentration of glucose is sufficient for the heterotrophic growth of many microalgae. However, understanding of the adverse effects of high concentration of glucose on microalgal growth is rather limited. There are at least three underlying mechanisms to be investigated: (a) excessive glucose may interact with the symporter and inhibit uptake of glucose directly; (b) high glucose level could cause oxidative stress in microalgal cells as it does in mammalian cells; (c) inhibitory metabolic by‐products (e.g., ethanol or acetate) caused by Crabtree effect under high glucose concentration may secrete into the growth medium.

This study is not only a proof of concept that combining heterotrophic and photoautotrophic cultivation for oil production on a pilot scale is economically feasible, but also demonstrated that microalgal cells grown under heterotrophic conditions were conferred a superiority in terms of lipid production as compared to the photoautotrophic cells. Heterotrophically grown cells of the green alga *Haematococcus pluvialis* were found to be susceptible to excessive light, which is attributable to the impairment of photosynthetic machinery in the dark (Zhang et al., 2016). However, *S. acuminatus* cells from the high‐cell‐density heterotrophic culture acclimated to the photoautotrophic conditions, while accumulating oils at a higher rate than the photoautotrophically grown cells. Similar with the findings of this study, a number of previous studies have observed that the heterotrophic *Chlorella* cells showed higher growth rate and lipid productivity than the photoautotrophic cells when they are subjected to the same stress conditions (Han et al., 2012; Zheng et al., 2012).

Intriguingly, when compared to the photoautotrophic cells, enhancement in the growth rate of heterotrophic cells is coincident with the reduction in the cellular content of chlorophyll, implying that these cells could possess truncated light‐harvesting antennae. It is well‐known that this alteraion led to increased light penetration in high‐cell‐density‐culture, less likelihood of photoinhibition, and reduced energy loss as heat (Melis, 2009). Our intuitive speculation, however, remains to be tested by more detailed biochemical and physiological analysis. If this is a fact, it indicates glucose could be used to tune the composition and structure of photosystems as to improve the growth capability without changing the genetic make‐up of a given algal species.

## CONFLICT OF INTERESTS

The authors declare that there are no conflict of interests.

## AUTHOR CONTRIBUTIONS

H. J., H. Z. participated in the design and performance of experiments, data collection and analysis, and manuscript writing. H. J., H. Z., Z. W. Z., K. P. L., G. L. H., and W. H. C. participated in the heterotrophic cultivation. H. Z., Q. X. participated in the autotrophic cultivation. C. W. Z. provided the experiment microalgae. G. L. H. participated in TE analysis. D. X. H. and Q. H. participated in design of experiments and manuscript writing. H. J. and H. Z. contributed equally.

## Supporting information

Supporting information
