# Supplementary material for: Ultrahigh‐cell‐density heterotrophic cultivation of the unicellular green microalga Scenedesmus acuminatus and application of the cells to photoautotrophic culture enhance biomass and lipid production
Source: Biotechnol Bioeng. 2019 Nov 12;117(1):96–108. doi: 10.1002/bit.27190 (PMC6916281; doi:10.1002/bit.27190)
Supplement: Supplementary file 1 — Supporting information [file BIT-117-96-s001.docx]

**Supplementary materials**

Ultrahigh-cell-density heterotrophic cultivation of the unicellular green microalga *Scenedesmus acuminatus* and application of the cells to photoautotrophic culture enhance biomass and lipid production

Hu Jin^1, †^, Hu Zhang^1, 2, †^, Zhiwei Zhou^3^, Kunpeng Li^1^, Guoli Hou^1^, Quan Xu^1, 2^, Wenhua Chuai^4^, Chengwu Zhang^3^, Danxiang Han^1, 5*^, Qiang Hu^1, 4, 5, 6*^

^1^Center for Microalgal Biotechnology and Biofuels, Institute of Hydrobiology, Chinese Academy of Sciences, Wuhan 430072, China

^2^College of life Sciences, University of Chinese Academy of Sciences, Beijing 100049, China

^3^Research Center of Hydrobiology, Jinan University, Guangzhou 510632, China

^4^Microalgae Biotechnology Center, SDIC Biotech Investment Co., LTD., State Development & Investment Corp., Beijing 100142, China

^5^Key Laboratory of Algal Biology, Institute of Hydrobiology, Chinese Academy of Sciences, Wuhan, Hubei 430072, China

^6^Beijing Key Laboratory of Algae Biomass, SDIC Biotech Investment Corporation, Beijing 100142, China

*Corresponding authors: Danxiang Han (danxianghan@ihb.ac.cn) and Qiang Hu (huqiang@ihb.ac.cn)

^†^Hu Jin and Hu Zhang contributed equally.

Figure S1 Comparison of DO and agitation rate during the whole fermentation under 30 ºC (a) and 35 ºC (b) cultivation (Red line: DO; Blue line: Agitation rate).

Figure S2 Temperature (a), light intensity (b) and pH (c) of the pilot-scale cultures in the tubular photobioreactors for lipid production with the seeds from heterotrophic (black line) and autotrophic (red line) cultures.


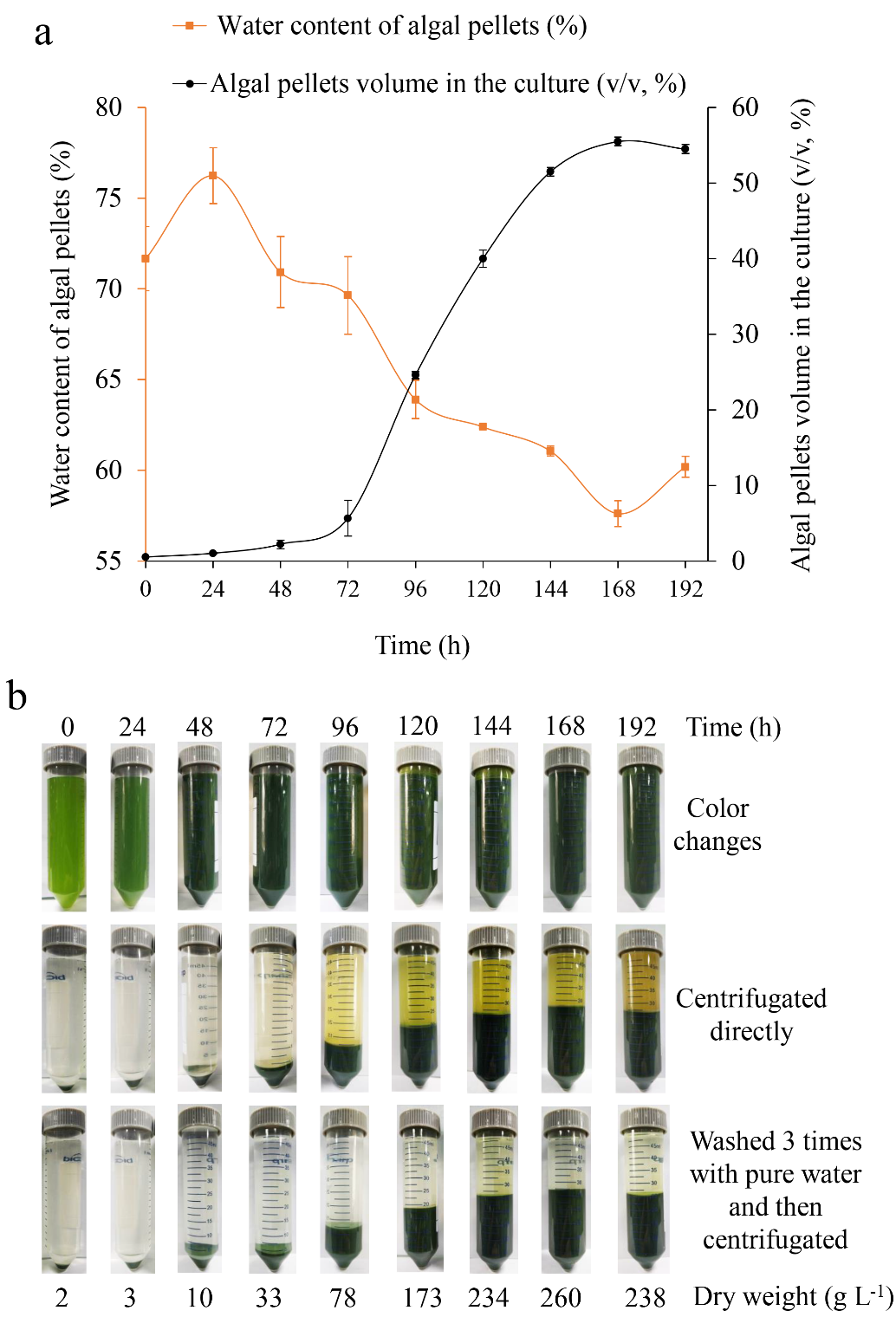


Figure S3 Heterotrophic culture of *S. acuminatus* in fermentor. (a) Changes of algal pellets volume in the culture (Black line) and water content of algal pellets (Red line) during fermentation, (b) Changes of culture color and algal pellets volume (the culture suspension was centrifugated at 4000 g and 10 min) during fermentation.

Table S1 Basic parameters of heterotrophic cultivation.

| **No.** | **Items** | **Unit** | **1,000 tons/year** | **10,000 tons/year** |
| --- | --- | --- | --- | --- |
| 1 | Fermentor size | m^3^ | 120 | 200 |
| 2 | Fermentor number |  | 3 | 8 |
| 3 | Harvest volume rate | % (v/v) | 80 | 80 |
| 4 | Ave. biomass conc. | g L^-1^ | 200 | 200 |
| 5 | Culture period | d | 10 | 7 |
| 6 | Annual operating time | d | 300 | 300 |
| 7 | Collapse rate | % | 10 | 10 |
| 8 | Annual production batches |  | 66 | 377 |
| 9 | Glucose conversion rate | % | 55 | 55 |

Table S2 Basic parameters of open pond and tubular photobioreactors.

| **No.** | **Items** | **Unit** | **1, 000 tons/year** | | **10, 000 tons/year** | |
| --- | --- | --- | --- | --- | --- | --- |
|  |  |  | **Tubular photobioreactor** | **Open pond** | **Tubular photobioreactor** | **Open pond** |
| 1 | Culture period | d | 10 | 10 | 10 | 10 |
| 2 | Annual operating time | d | 300 | 300 | 300 | 300 |
| 3 | Initial biomass conc. | g L^-1^ | 0.2 | 0.1 | 0.2 | 0.1 |
| 4 | Harvest biomass conc. | g L^-1^ | 2 | 0.8 | 2 | 0.8 |
| 5 | Size of reactor |  | L-125m | 250m×20m×0.3m | L-125m | 250m×20m×0.3m |
| 6 | Single reactor volume | m^3^ | 98 | 1,000 | 98 | 1,000 |
| 7 | Collapse rate | % | 5 | 15 | 5 | 15 |
| 8 | Reactor number |  | 179 | 50 | 1788 | 491 |
| 9 | Annual yield | t | 1,052 | 1,176 | 10,526 | 11,764 |
